# Changes in the Tumor Immune Microenvironment during Disease Progression in Patients with Ovarian Cancer

**DOI:** 10.3390/cancers12123828

**Published:** 2020-12-18

**Authors:** Marie Christine Wulff Westergaard, Katy Milne, Magnus Pedersen, Thomas Hasselager, Lars Rønn Olsen, Michael S. Anglesio, Troels Holz Borch, Mia Kennedy, Gillian Briggs, Stacey Ledoux, Caroline Kreuzinger, Isabel von der Decken, Marco Donia, Dan Cacsire Castillo-Tong, Brad H. Nelson, Inge Marie Svane

**Affiliations:** 1 National Center for Cancer Immune Therapy (CCIT-DK), Department of Oncology, Copenhagen University Hospital, 2730 Herlev, Denmark; marie.christine.wulff.westergaard@regionh.dk (M.C.W.W.); magnus.pedersen.01@regionh.dk (M.P.); troels.holz.borch@regionh.dk (T.H.B.); marco.donia@regionh.dk (M.D.); 2 Deeley Research Centre, BC Cancer, Victoria, BC V8R 6V5, Canada; kmilne@bccrc.ca (K.M.); mk3nnedy@telus.net (M.K.); gillian.briggs11@gmail.com (G.B.); staceyledoux29@gmail.com (S.L.); bnelson@bccrc.ca (B.H.N.); 3 Department of Pathology, Copenhagen University Hospital, 2730 Herlev, Denmark; thomas.hasselager@regionh.dk; 4 Department of Health Technology, Technical University of Denmark, 2800 Kgs Lyngby, Denmark; lronn@dtu.dk; 5 Genomic Medicine, Copenhagen University Hospital, 2100 Copenhagen, Denmark; 6 Department of Obstetrics and Gynaecology, University of British Columbia, Vancouver, BC V5Z 1L3, Canada; m.anglesio@ubc.ca; 7 Translational Gynecology Group, Department of Obstetrics and Gynecology, Comprehensive Cancer Center, Medical University of Vienna, 1090 Vienna, Austria; caro.kreuzinger@hotmail.com (C.K.); isabel.vonderdecken@unifr.ch (I.v.d.D.); dan.cacsire-castillo@meduniwien.ac.at (D.C.C.-T.); 8 Department of Medical Genetics, University of British Columbia, Vancouver, BC V5Z 1L3, Canada

**Keywords:** ovarian cancer, tumor microenvironment, immune checkpoints, TILs, multicolor immunohistochemistry (IHC), NanoString, adaptive immune resistance

## Abstract

**Simple Summary:**

Immunotherapy has been a successful treatment for many cancers. However, no immunotherapy treatment has been approved for ovarian cancer due to low efficacy in this patient group. This study investigated the cellular and molecular changes from primary ovarian tumors, at the time of diagnosis, to recurrence, where the disease returns after surgery and chemotherapies. Here we examined the immune contexture to better understand subdued responses to immunotherapy and identify additional, potentially complimentary, therapeutic targets. Indications of the development of adaptive immune resistance during disease progression were observed, with increases in immune and stromal cell infiltration accompanied by the expression of immune suppressive markers. We observed high gene expression of the immune checkpoint genes *LAG3* and *HAVCR2* (TIM3) in most tumors and increased expression of the immune checkpoint genes *TIGIT* and *CTLA4* in recurrent tumors, compared to the primaries. These markers may be potential candidates for immunotherapy targeting in ovarian cancer.

**Abstract:**

Anti-PD1/PDL1 therapy has proven efficacious against many cancers but only reached modest objective response rates against recurrent ovarian cancer. A deeper understanding of the tumor microenvironment (TME) may reveal other immunosuppressive mechanisms that warrant investigation as immunotherapeutic targets for this challenging disease. Matched primary and recurrent tumors from patients with high-grade serous ovarian carcinoma (HGSC) were analyzed by multicolor immunohistochemistry/immunofluorescence for the presence of T cells, B cells, macrophages, and for the expression of immunosuppressive and HLA molecules. Cancer- and immune-related gene expression was assessed by NanoString analysis. Recurrent tumors showed increased infiltration by immune cells, displayed higher expression of PDL1, IDO, and HLA molecules, and contained more stromal tissue. NanoString analysis demonstrated increased expression of gene signatures related to chemokines and T cell functions in recurrent tumors. The ovarian tumors showed high gene expression of *LAG3* and *HAVCR2* (TIM3) and enhanced levels of *TIGIT* and *CTLA4* in recurrent tumors compared to primary tumors. The majority of HGSC developed into a more inflamed phenotype during progression from primary to recurrent disease, including indications of adaptive immune resistance. This suggests that recurrent tumors may be particularly sensitive to inhibition of adaptive immune resistance mechanisms.

## 1. Introduction

Metastatic ovarian cancer is the most lethal gynecological cancer [1]. The standard of care is currently platinum-based chemotherapy and debulking surgery. Even though most patients initially respond to treatment, the majority will relapse, resulting in a 5-year survival rate of only 47% [2,3,4]. Studies of the tumor microenvironment (TME) over the past two decades has deepened our understanding of tumor progression/evolution and highlighted the importance of immune cells [5]. In particular, the presence of tumor-infiltrating CD8+ T cells in primary high-grade serous ovarian tumors is strongly associated with prolonged overall survival (OS) and disease-specific survival (DSS) [6,7,8,9,10,11].

Moreover, tumor-infiltrating B cells and plasma cells are associated with increased survival [11,12]. However, the TME is also replete with immune suppressive factors, including programmed cell death 1 (PD1) and its main ligand, PD-ligand 1 (PDL1) [13,14,15,16]. Furthermore, a high abundance of tumor-associated macrophages (TAMs) has been shown associated with a poor prognosis [17]. This could be related to the strongly immunosuppressive and protumorigenic nature of TAMs [18].

PD1 and PDL1 can be therapeutically targeted with clinically approved checkpoint inhibitor (CPI) monoclonal antibodies, which have proven highly effective in diverse cancer subtypes [19,20,21]. However, in ovarian cancer, the objective response rate to CPI therapy is only 5.8–15% [22,23,24,25,26], highlighting the need for other immunotherapy strategies. In mouse models, other checkpoints, such as lymphocyte activation gene 3 (LAG3) and T cell immunoglobulin and mucin domain 3 (TIM3), have been investigated as new targets for CPI treatment. Promising results were observed when treating the mice with antibodies targeting either LAG3 or TIM3 in combination with other immunotherapy treatments [27,28], encouraging further investigation in patients with ovarian cancer. Another approach could include inhibition of the immunosuppressive enzyme indoleamine 2,3-dioxygenase (IDO). IDO is highly expressed in many cancers, including approximately half of ovarian cancers [29,30,31]. High expression of IDO in the TME promotes tumor progression [29], providing a rationale for the therapeutic targeting of this enzyme [32]. One trial was carried out treating patients with ovarian cancer with IDO inhibitor, though a limited response was observed [33].

To improve immunotherapy and expand the target options for ovarian cancer, a better understanding of how the TME changes during disease progression from primary to recurrent disease is needed. To this end, we evaluated major immune cell subsets and regulatory factors in matched primary and recurrent tumor samples from a high grade serous ovarian carcinoma (HGSC) patient cohort treated with standard of care between the primary and recurrent tumor resection.

## 2. Results

### 2.1. Patient Characteristics

We studied matched primary and recurrent tumor samples from 9 HGSC patients who were enrolled in a phase I clinical trial of adoptive T cell therapy (ACT) after recurrence; however, the recurrent samples were obtained before the patients received ACT. Clinicopathological and treatment information is provided in Table S1. All patients had stage III or IV disease and most received frontline treatment with carboplatin/taxane-based chemotherapy; two patients also received bevacizumab. Subsequent chemotherapy prior to collection of the recurrent samples included agents such as carboplatin, taxanes, gemcitabine, caelyx, and bevacizumab. The time between primary and recurrent tumor collection ranged from 9–171 months (mean = 62.7 months).

### 2.2. Tumor Recurrence is Associated with Higher Levels of Immune Cell Infiltration

Primary and recurrent tumors were evaluated by multicolor immunohistochemistry (IHC) to detect T cells (CD3/CD8/TIA1), B cells/plasma cells (CD20/CD79a) and TAMs (CD163/PDL1).

**Figure 1:**
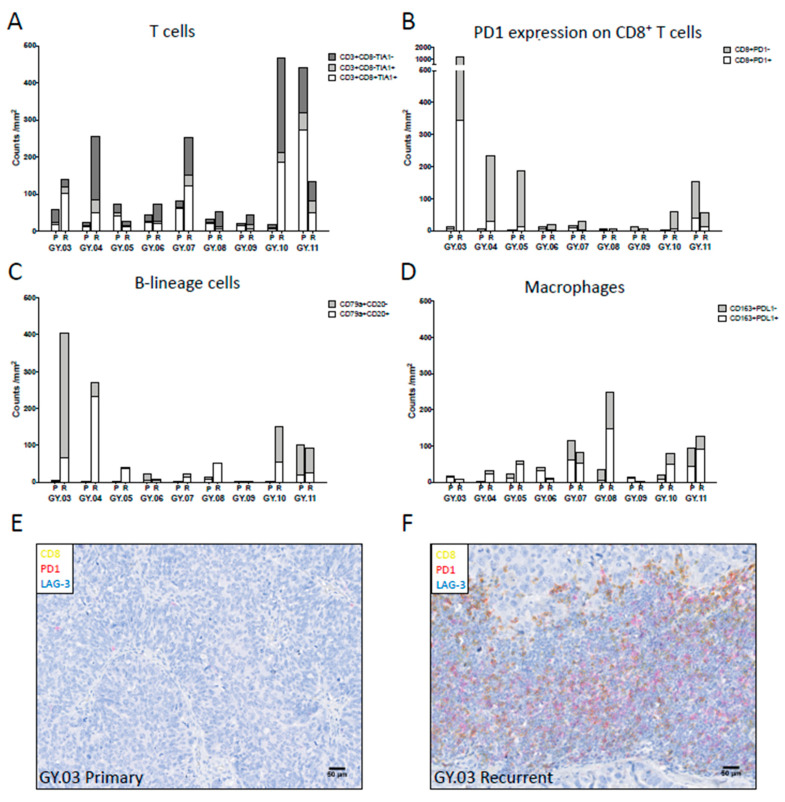
The Immune infiltration in the tumor microenvironment (TME) of the primary (P) and recurrent (R) ovarian tumor samples was analyzed using multicolor IHC analysis. (**A**) T cell panel illustrating cytotoxic CD8+ T cells (CD3+CD8+TIA1+) in white, cytotoxic CD4+ T cells (CD3+CD8-TIA1+) in light grey and helper CD4+ T cells (CD3+CD8-TIA1−) in dark grey. (**B**) Exhaustion panel illustrating exhausted/antigen-specific CD8+ T cells (CD8+PD1+) in white and PD-1 negative CD8+ T cells (CD8+PD1−) in light grey. (**C**) B cell panel illustrating B cells (CD79a+CD20+) in white and plasma cells (CD79a+CD20−) in light grey. (**D**) Macrophage panel illustrating immunosuppressive subset (CD163+PDL1+) in white and PDL1 negative subset (CD163+PDL1−) in light grey. (**E**,**F**) Example of PD1 and LAG3 expression on CD8+ T cells in primary (**E**) and recurrent (**F**) tumor tissue in patient GY.03. CD8 in yellow PD1 in red and LAG3 in blue.

In general, recurrent tumor samples exhibited denser immune cell infiltrates compared to primary samples (Figure 1); only one patient (GY.11) showed decreased immune cell infiltration in the recurrent sample and one (GY.05) showed a mixture of increased and decreased infiltrated. In particular, recurrent tumors showed increased cytotoxic CD8+ T cells (CD3+CD8+TIA1+) and putative helper CD4+ T cells (CD3+CD8-TIA1−) (Figure 1A,E,F). The change in CD8+ T cell infiltration was mostly due to a higher number of PD1-negative CD8+ T cells (Figure 1B). Virtually no LAG3 expression was observed in any of the samples.

In 6/9 patients, B cells (CD79a+CD20+) and plasma cells (CD79+CD20−) showed greater densities in recurrent tumors (Figure 1C). Indeed, three patients (GY.03, GY.04 and GY.10) showed massive increases in both B cells and plasma cells, which were associated with dramatic increases in T cell subsets as well.

The density of TAMs (CD163+) increased at recurrence in 5/9 patients (Figure 1D). These patients were not necessarily those with large increases in T cells and B-lineage cells. In primary tumors, 55% of CD163 TAMs expressed PD-L1, and this proportion increased to 65% in recurrent tumors (Figure S1).

### 2.3. HLA Expression Is Upregulated in Recurrent Tumors

**Figure 2:**
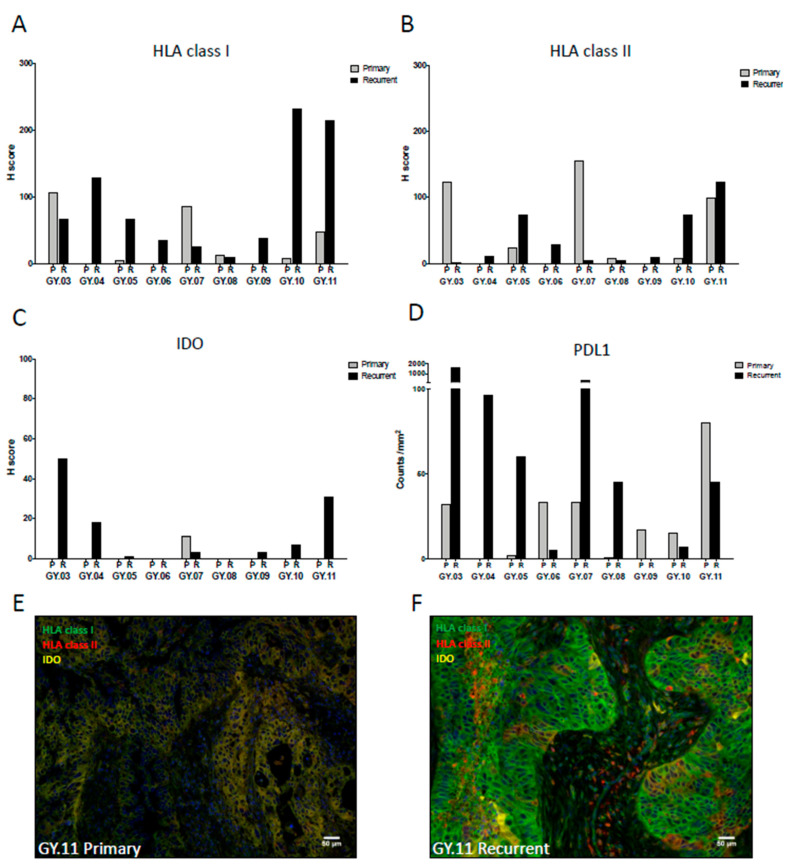
The expression of immune-modulating molecules on tumor cells in the TME of the primary (P) and recurrent (R) tumor samples were analyzed using multispectral immunohistochemistry (IHC) analysis or immunofluorescent IHC. (**A**–**D**) expression in primary tumor is presented in light grey and expression in recurrent tumor is presented in black. (**A**) Expression of HLA class I in the epithelial compartment of the TME. (**B**) HLA class II in the epithelial compartment of the TME. (**C**) Expression of IDO in the epithelial compartment of the TME. (**D**) Expression of PDL1 (CD163-PD1-PDL1+). (**E**,**F**) Example of HLA and indoleamine 2,3-dioxygenase (IDO) expression in primary (**E**) and recurrent (**F**) tumor tissue in patient GY.11. HLA class I in green, HLA class II in red, and IDO in yellow.

T cell-mediated killing is dependent on TCR-based recognition of HLA: epitope complexes on cancer cells. Accordingly, HLA downregulation is a well-established immune resistance mechanism. Surprisingly, cancer cells showed upregulated expression of both HLA class I and II on recurrent tumor samples in 6/9 patients. In three of these cases (GY.04, GY.06, and GY.09), primary tumors were completely negative for both HLA molecules, yet recurrent tumors were clearly positive (Figure 2A,B,E,F). In 3/9 patients (GY.03, GY.07, and GY.08), recurrent tumors showed HLA downregulation, although none became completely negative.

### 2.4. Immunosuppressive Molecules Increase in Some Patients While Decreasing in Others

Only 1/9 primary tumor sample was positive for IDO. Strikingly, at recurrence, IDO was detected in 7/9 tumor samples (Figure 2C,E,F). Some of the highest increases were seen in patients GY.03 and GY.04, which also exhibited large increases in T cells and B cells. However, patient GY.11 showed higher levels of IDO expression in spite of decreased T cell infiltration.

In addition to evaluating PDL1 expression on macrophages (Figure 1C), we assessed expression on CD163-negative cells, the vast majority of which appeared to be cancer cells. PDL1 expression by cancer cells increased dramatically in 5/9 recurrent tumor samples, the majority of which also showed increases in T cells and B cells/plasma cells (Figure 2D). Expression of PDL1 decreased significantly in the remaining 4/9 cases (GY.06, GY.09, GY.10, and GY.11) with no apparent association with changes in T cells or B cells/plasma cells. The observed trend of large increases in the expression of the two immunosuppressive molecules from primary to recurrent tumor samples is accompanied by increases in T cells and B cells in the majority of the patients. This could indicate development of adaptive immune resistance during disease progression.

### 2.5. Increased Stromal Infiltration in the TME

The area of the stromal compartment of the TME was quantified in the IHC analysis. Recent studies have indicated that tumor stroma can have important immunosuppressive activity [34,35,36]. In five of the patients, an apparent increase in the proportion of stromal tissue was observed from primary to recurrent TME (Figure S2). Two patients had a similar proportion, and two patients had a decrease in the proportion of stromal tissue in the TME from primary to recurrent tumor samples (Figure S2A,B). Interestingly, GY.11 displayed a decrease in the proportion of stromal tissue, matching the decrease in T cells and PDL1 expression observed in this patient (Figure S2A).

### 2.6. Higher Cancer- and Immune-Related Gene Expression in Recurrent Tumors

**Figure 3:**
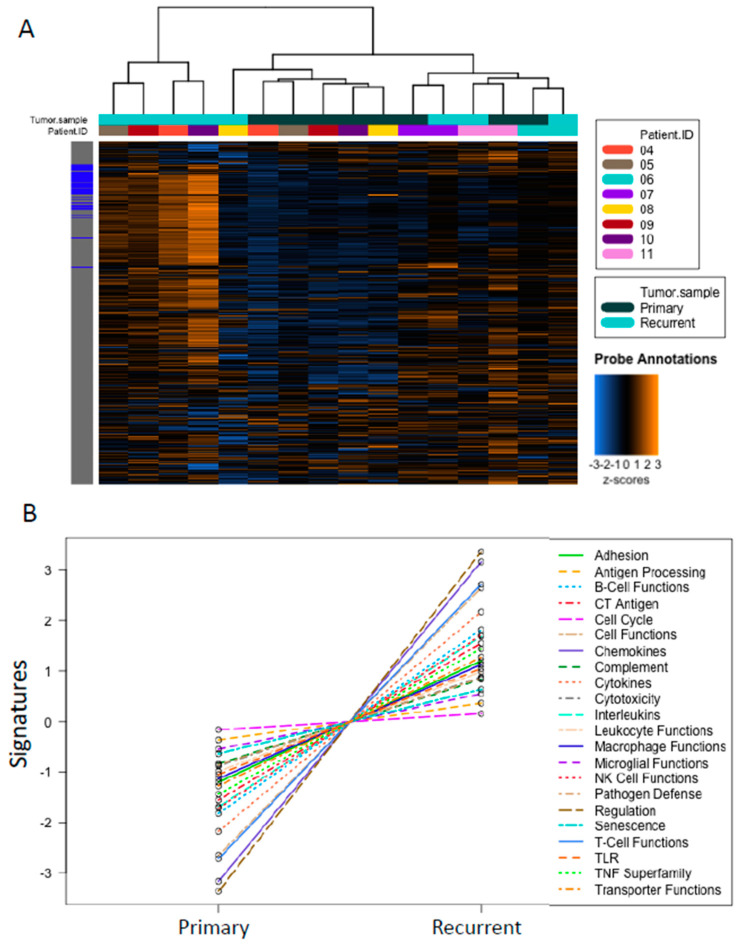
NanoString analysis of primary vs. recurrent tumor samples. (**A**) Heatmap showing all genes analyzed with NanoString technology. Samples are clustered together based on expression patterns. Gene expression levels are illustrated with a Z score. Orange indicates high expression; blue indicates low expression. (**B**) Illustrating the change in the pathway signatures from primary to recurrent tumors using nSolver software.

NanoString analysis of a panel of 770 cancer- and immune-related genes was performed on primary and recurrent tumor samples from eight patients. Data were normalized to 38 housekeeping genes. Overall, higher expression of the genes in this panel was observed in recurrent compared to primary tumor samples (Figure 3A). In particular, genes involved in chemokine activity and T cell function showed the greatest increases (Figure 3B).

This pattern was particularly evident for patients GY.04, GY.05, GY.08, GY.09, and GY.10 whose primary tumors formed a single cluster that was distinct from the cluster formed by their recurrent tumors (Figure 3A). In contrast, for the remaining three patients, primary and recurrent tumors clustered in a pairwise manner, indicating relatively fewer gene expression changes occurred between primary and recurrent disease. These patterns largely mirrored the changes in stromal content (Figure S2A) more so than immune cell patterns (Figure 1 and Figure 2).

### 2.7. Indications of Adaptive Immune Resistance

**Figure 4:**
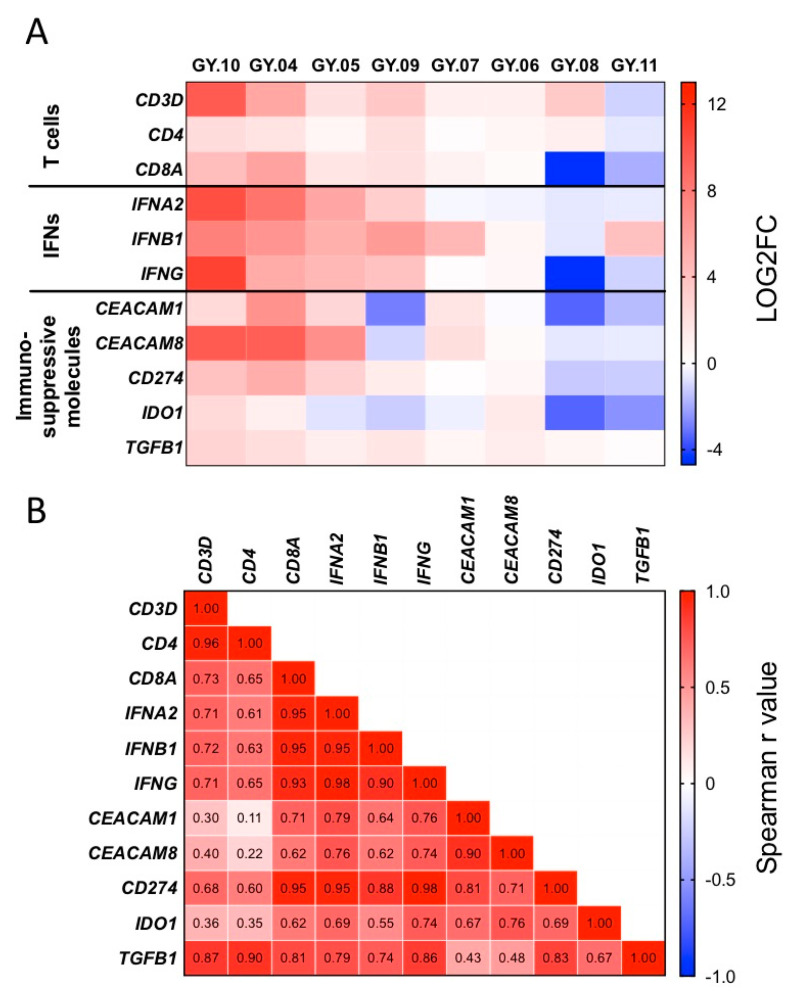
NanoString analysis of primary vs. recurrent tumor samples. (**A**) Heatmap showing the log2 fold change values of the expression of selected genes from the primary and recurrent tumor samples analyzed with NanoString technology. Red indicates high expression and blue indicates low expression. (**B**) Spearman correlation matrix with r values. Red indicates a strong positive correlation, blue indicates a strong negative correlation and white indicates no correlation.

Using primary tumor as the baseline, the change in gene expression levels of T cell markers, interferons and immunosuppressive molecules were plotted in a heatmap to provide a graphical overview (Figure 4A). In 4/8 matched tumor samples (GY.04, GY.05, GY.09, and GY.10), recurrent tumors showed higher expression levels of genes related to interferons of T cells. In 6/8 cases, increased expression of CEACAM and PDL1 genes was seen (Figure 4A). The presence of a seemingly active T cell infiltrate paired with disease progression suggest underlying adaptive immune resistance mechanisms. Spearman correlation analyses supported these findings, showing strong positive correlations between the gene expression changes of T cell genes, interferons and the immunosuppressive molecules PDL1, TGFB1 and to some degree IDO and CEACAMs (Figure 4B).

There was some conformity between the gene expression analysis and the observations from the IHC with the changes in T cell infiltration from primary to recurrent tumor being accompanied by similar changes in T cell-related gene expression in e.g., patients GY.04, GY.09, and GY.10, and GY.11 (Figure 1A and Figure 4A). However, the detection of IDO and PDL1 by IHC did not correlate with gene expression.

### 2.8. Top Upregulated Genes, CD36 and CD44

**Figure 5:**
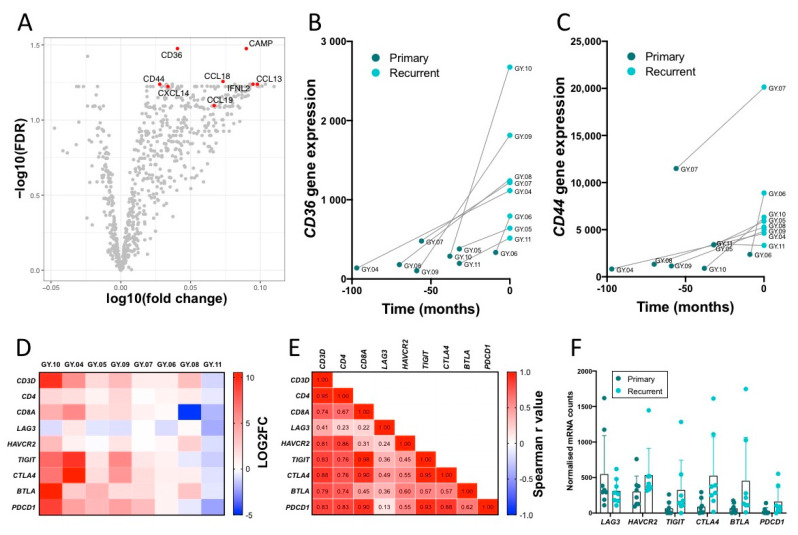
NanoString analysis of top upregulated genes in recurrent tumor compared to primary tumor samples. (**A**) Volcano plot showing Log2 fold change and the false discovery rate (FDR) using the primary tumor samples as the baseline. Illustration of expression of *CD36* (**B**) and *CD44* (**C**) over time (months from primary to recurrence). Statistical analysis was performed with a Wilcoxon test. (**D**–**F**) shows the expression of T cell marker genes and immune checkpoint genes. (**D**) Heatmap showing the log2 fold change values of the expression of selected genes from the primary and recurrent tumor samples. Red indicates high expression and blue indicates low expression. (**E**) Spearman correlation matrix with r values. Red indicates a strong positive correlation, blue indicates a strong negative correlation and white indicates no correlation. (**F**) Gene expression levels of immune checkpoints in primary and recurrent tumors. Statistical significance between expression levels in primary and recurrent samples was tested using multiple Wilcoxon tests. No significant changes were found with adjusted p values, false discovery rate (FDR), *LAG3*: *p* = 0.252, *HAVCR2*: *p* = 0.252, *TIGIT*: *p* = 0.224, *CTLA4*: *p* = 0.118, *BTLA*: *p* = 0.118, *PDCD1*: *p* = 0.224.

A volcano plot of the gene expression revealed a small number of highly upregulated genes, including genes encoding the proteins; IFNL2, CAMP, two multifunctional cell surface proteins CD36 and CD44, and the four chemokines: CCL13, CCL18, CCL19, and CXCL14 (Figure 5A).

A further illustration of how the gene expression level changed over time was made for CD36 and CD44 by plotting expression levels measured by NanoString against the time gap between primary and recurrent tumor samples (Figure 5B,C). These illustrations show that the observed upregulation in the volcano plots is due to a general increase in all patients except patient GY.11.

CD36 can be expressed on several different cell types, including both lymphocytes, cancer cells, and fibroblasts. To interpret whether the observed higher expression levels were due to increased lymphocyte infiltration, R2 correlation tests of *CD36* vs. *CD3D*, *CD36* vs. *EPCAM* and *CD36* vs. *COL1A2* were performed (R2 = 0.553, R2 = 0.358, and R2 = 0.06 respectively), which suggested that CD36 was mainly, but not exclusively expressed on T cells (Figure S3). Furthermore, there was a stronger correlation of *CD36* vs. *FOXP3* than *CD36* vs. *CD8A* and *CD36* vs. *CD4*, indicating CD36 expression by Tregs (Figure S3D,F).

### 2.9. Infiltrating T Cells and Immune Checkpoint Expression

With the Nanostring analysis, we further looked at the gene expression of several immune checkpoint genes in the TME. The expression increased in the majority of the patients (Figure 5D). The expression of immune checkpoint genes correlated strongly with the expression of T cell markers (Figure 5E). A strong correlation between the Log2 fold change of the T cell gene *CD3D* and the immune checkpoint molecules genes, *CTLA4*, *PDCD1* (PD1), *TIGIT*, *HAVCR2* (TIM3), and *BTLA* was observed in the TME (spearman r values: 0.88, 0.83, 0.83, 0.81, and 0.79, respectively) whereas *LAG3* only showed a weak correlation (spearman r value: 0.41). BTLA and HAVCR2 correlated stronger to CD4+ T cells (*CD4*), whereas *TIGIT* and *CTLA4* correlated stronger to CD8+ T cells (*CD8A*) (Figure 5E). However, when looking at the expression level, we observed relatively high expression of *LAG3* and *HAVCR2* in both primary and recurrent tumor and a trend of greater expression for *TIGIT*, *CTLA4*, *BTLA,* and *PDCD1* in recurrent tumor tissue, compared to primary tumor tissue (NS) (Figure 5F). In addition to the checkpoint molecules, we also observed a strong correlation between *CD3D*, the lymphocyte activation marker *TNFRSF9* (CD137), and to genes encoding granzymes (Figure S4).

### 2.10. Validation of Findings in a Separate Cohort

To confirm our findings in the GY patient cohort, we used previously published RNA sequencing data from a cohort of patients (n = 66) with matched primary and recurrent ovarian tumors [37].

**Figure 6:**
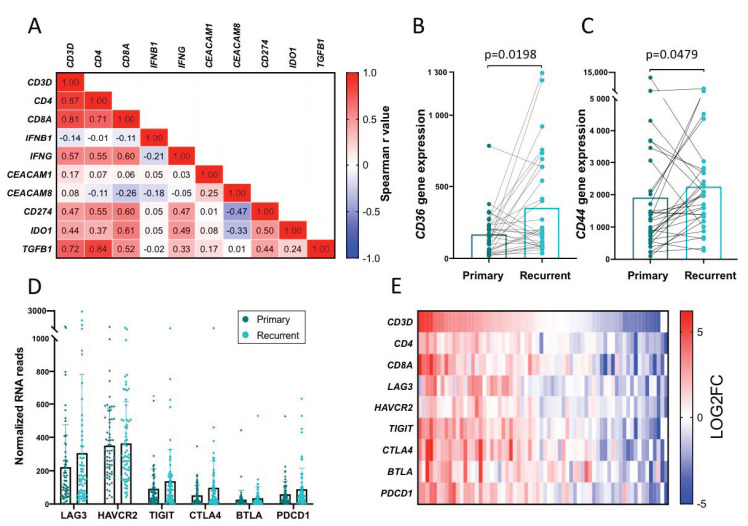
Validation of findings in an independent cohort of 66 patients with RNA seq data from primary and recurrent tumors described in Kreuzinger et al. [37] (**A**) Spearman correlation matrix with r values, from log2 fold change values of the expression of selected genes from the primary and recurrent tumor samples. Red indicates a strong positive correlation, blue indicates a strong negative correlation and white indicates no correlation. Validation of gene expression of *CD36* (**B**) and *CD44* (**C**). Figure (**B**,**C**) show patients with an increase in the T cell infiltration, with a log2 fold change >0.5 of the *CD3D* gene from primary to recurrent tumors. Statistical analysis was performed with a Wilcoxon test. (**D**) Gene expression levels of immune checkpoints in primary and recurrent tumors. Statistical significance between expression levels in primary and recurrent samples was tested using multiple Wilcoxon tests. No significant changes were found with adjusted p values, false discovery rate (FDR), *LAG3*: *p* > 0.999, *HAVCR2*: *p* > 0.999, *TIGIT*: *p* = 0.100, *CTLA4*: *p* = 0.057, *BTLA*: *p* = 0.100, *PDCD1*: *p* = 0.100. (**E**) Heatmap showing the log2 fold change values of the expression of selected genes from the primary and recurrent tumor samples. Red indicates high expression and blue indicates low expression.

A heatmap illustrating the Log2 fold change of the expression of selected genes from primary to recurrent tumor confirmed the pattern that an increase in T cell gene expression is associated with an increase in *IFNG* gene expression and gene expression of immunosuppressive molecules such as *CD274* (PDL1), *IDO1,* and *TGFB1*—indicative of adaptive immune resistance (Figure S5A). This was further confirmed by a spearman correlation test, illustrated by a heatmap (Figure 6A).

The increase of *CD36* and *CD44* gene expression over time observed in our cohort (Figure 5B,C) was not confirmed in the cohort from Kreuzinger et al. (Figure S5B,C). Though, when only looking at the patients with a log2 fold increase >0.5 of the *CD3D* gene, a significant increase in the *CD36* and *CD44* gene expression at the tumor site was revealed (*p* = 0.0198 and *p* = 0.0479, respectively, Wilcoxon ranked *t*-test) (Figure 6B,C)—indicating that these genes were upregulated in patients with increased T cell infiltration.

We further validated our findings of the immune checkpoint gene expression and the correlation to T cells in the cohort from Kreuzinger et al. We confirmed the high gene expression levels of *LAG3* and *HAVCR2* in both primary and recurrent tumor tissue and a trend towards increased gene expression of *TIGIT*, *CTLA4,* and *PDCD1* in recurrent tumor compared to primary tumor (Figure 6D). Furthermore, the correlation of T cell genes with the immune checkpoint genes observed in our cohort (Figure 5E) was also present in this larger cohort with especially a strong correlation with *TIGIT*, *CTLA4,* and *PDCD1* (Figure 6E and Figure S5D).

### 2.11. Expression of Immune Checkpoints on In Vitro Expanded TILs from Recurrent Tumor

We investigated the expression of immune checkpoints on TILs from the recurrent tumor also used as the infusion product for treating the patients with ACT. On CD8+ T cells, we observed higher levels of LAG3, TIM3, and TIGIT compared to PD1 expression (*p* = 0.0195, *p* = 0.0547, and *p* = 0.0039, respectively, Wilcoxon matched pairs signed rank test) (Figure S6), indicating these immune checkpoints to be potential targets for reinvigorating the cytotoxic and possibly tumor-specific T cells.

## 3. Discussion

In the era of modern immunotherapy, an increasing focus has been on understanding the evolution and dynamics of the TME in cancer. This study investigated the changes in immune infiltration, expression of immunosuppressive molecules and gene expression levels of immune-related genes from primary tumor to recurrent HGSC tumors in matched samples. Between sampling, patients had received adjuvant treatment and at least one line of chemotherapy in the metastatic setting. The majority of the recurrent tumor samples were more infiltrated by lymphocytes and macrophages compared to the matched primary tumor sample. Furthermore, the protein expression level of the immunosuppressive molecule IDO, density of PDL1 positive cells and expression level of genes related to T cell function, chemokines, and cytokines were generally higher in the recurrent tumor samples across most of the examined patients. Even so, we found diverse patterns of changes in these parameters, indicating tumor heterogeneity and patient-specific mechanisms of progression.

Higher PDL1 levels in recurrent tumor tissue have also reported by Aust et al. [15] in a similar comparison of primary and recurrent ovarian tumor tissue. Furthermore, they noted that HLA class I genes were negatively correlated with PDL1 protein expression and speculated in different escape strategies. However, such negative correlation between protein levels of PDL1 and HLA class I genes was not found in the present data.

Another recent study did not observe a change in the PDL1 levels from the primary to the recurrent ovarian tumor [38], and they found the CD8+ T cell infiltration to increase and decrease in an equal number of patients. This underlines the need for a larger cohort of patients to be investigated before the importance and frequency of different immune escape mechanisms is fully elucidated and it also illustrates how cancer evolution varies in individual patients.

Previous studies have suggested IDO plays a role in the progression of ovarian cancer [29,39,40]. In primary tumors, strong expression levels of IDO has been reported for the majority of patients with ovarian cancer and co-expression of IDO and PDL1 has been shown to correlate with high immune infiltration [41]. We detected the expression of IDO in only one out of nine primary tumor samples but, strikingly, in seven out of nine recurrent tumor samples, indicating IDO expression as an immune escape mechanism and a possible target. To this end, an IDO inhibitor was tested in a phase II clinical trial; however, no difference in efficacy was found compared to the control arm receiving tamoxifen (estrogen inhibitor) [33]. As IDO and PDL1 are often co-expressed along with frequent immune infiltration in recurrent tumor samples, a possible treatment strategy could be co-inhibition with anti-IDO and anti-PD-1/PDL1 antibodies or boosting the already present IDO- and/or PDL1-specific T cells with a vaccine [42].

For most of the patients in this study, the cancer evolved from a predominantly “cold” immune signature to a “hot” immune signature, according to the immune infiltration, IFN production and PDL1 expression as previously defined [43,44]. We were also able to expand tumor-reactive TILs from the recurrent tumor lesions, but nevertheless, these patients did not achieve clinical response with ACT [45]. This would be expected with a (primary) “hot” tumor as it is associated with a better clinical outcome and a better response rate to immunotherapy [43]. Further, we did not observe HLA class I depletion on the cancer cells, which is a well-known immune escape mechanism during tumor progression. In most cases, we instead observed an increased HLA expression at the protein level in the recurrent tumor, which should increase the immunogenicity of the tumors. This contradictory observation could indicate that stronger adaptive immune resistance mechanisms than the ones already described are at play, allowing the tumor to escape immune control.

We observed increased levels of tumor-infiltrating stroma in the majority of recurrent tumor samples. The tumor stroma has been shown to negatively influence immune activity in tumors by limiting T cell migration into the tumor core [46,47] but also by directly affecting T cell performance [48]. Gaining more insight into these mechanisms could reveal new therapeutic targets.

Due to our HGSC cohort only containing 9 patients, we validated our findings of specific patterns in a cohort of 66 patients with matched primary and recurrent ovarian tumors previously described by Kreuzinger and colleagues [37]. They divided the cohort into four groups based on their immune active or immune silent TME phenotypes from primary to recurrent tumor samples. ECM regulating genes were upregulated or downregulated in tumors with the active and silent immune status, respectively, which supports our findings of increased stroma in most of the patients experiencing increased immune infiltration in the recurrent tumor compared to the primary tumor.

The Kreuzinger cohort was examined using RNA sequencing on snap frozen tissue and our molecular analysis was performed using NanoString technology on formalin-fixed paraffin-embedded (FFPE) tissue. Even though we only compare patterns between the two cohorts and not expression levels, it has been well described that the NanoString technology gives robust data regardless of the sample preparation conditions (e.g., snap frozen and FFPE tissue) [49,50]. Further, the NanoString technology uses 38 housekeeping genes to ensure normalization of the tested samples among other strategies to correct for batch effects [51]. Therefore, we find it reasonable to use the Kreuzinger cohort as a validation cohort despite the two different analysis approaches. However, our cohort mostly represents patients with a tumor progression from “cold” to “hot” whereas the Kreuzinger cohort contains a more diverse pattern of tumor progression, which can explain why not all patterns are similar in the two cohorts.

We investigated gene expression patterns in the recurrent tumors vs. the primary tumors. Pathway signature analysis revealed that genes encoding chemokines and T cell functions were among the genes with the most considerable difference in the expression levels between primary and recurrent tumor samples. We found that the higher expression levels of T cell marker genes in the recurrent tumor compared to the primary tumor correlated with increased expression of several immune checkpoints and T cells activation genes. This observation, combined with the increase in granzymes, indicates that the T cells infiltrating the tumor are antigen-specific and activated. Furthermore, we observed high expression levels of the genes encoding LAG3 and TIM3 in the ovarian tumor tissue in both our cohort and the cohort from Kreuzinger et al., and indeed *LAG3* and *HAVCR2* (TIM3) were also expressed to a higher degree than PD1 on the in vitro expanded CD8+TILs from the recurrent tumor, indicating these checkpoint components play an inhibitory role in the TME of ovarian cancer and suggesting they may be attractive targets for new immunotherapies. However, for yet unknown reasons the LAG3 expression was not confirmed on a protein level with our IHC analysis. Both LAG3 and TIM3 have been investigated as targets for inhibitory monoclonal antibodies in ovarian mice bearing ovarian cancer models and showed promising results when used in combination with other immunotherapies [27,28]. To our knowledge no clinical trials treating patients with ovarian cancer with anti-LAG3 or anti-TIM3 monoclonal antibodies have been carried out. Thus, we have initiated the first clinical study treating patients with ovarian cancer with combinational therapy consisting of ACT, anti-PD1, anti-CTLA4, and anti-LAG3 (ClinicalTrials.gov identifier: NTC04611126). Additionally, the expression of the genes, *TIGIT*, *CTLA4*, and *PDCD1* were elevated in recurrent tumors compared to the primary. However, checkpoint inhibitor treatment targeting PD1 and CTLA4 has been tested in ovarian cancer patients with sparse response rates [22,25,26,52], leaving TIGIT as a remaining potential target for treating HGSC recurrence [53]. Two clinical trials treating cancer patients including patients with ovarian cancer with TIGIT inhibitors in combination with other immunotherapies have recently been initiated, however, no results have been reported yet [54,55].

In the current study, we also found that especially four chemokines were upregulated in the recurrent tumor compared to the primary tumor; CCL13, CCL18, CCL19, and CXCL14. They all attract a variety of immune cells including cells from both the innate and adaptive immune system, which is in line with our observation that most of the ovarian cancers evolve into a more inflamed tumor type during disease progression.

The NanoString analysis further revealed a limited number of genes that were dramatically increased in the recurrent tumors. Among others, the two multifunctional glycoproteins CD36 and CD44. Both molecules can bind to collagen, and it has been shown that high expression of CD36 is associated with lower recurrence-free survival in ovarian cancer [56]. The disadvantage of the NanoString analysis is the lack of information on which cells are expressing the specific markers. However, our correlation plots could indicate that CD36 is expressed on T cells and more by CD4+ T cells than CD8+ T cells, and the correlation with FoxP3 is consistent with expression by Tregs. A recent study identified CD36 expression on intratumoral Tregs as a central metabolic modulator for the Tregs to adapt to the lactic acid-enriched TME and thereby enhance their survival and suppressive activity [57]. The high increase in *CD36* gene expression following the increase in *CD3D* gene expression during disease progression combined with the lack of beneficial effect of the T cell infiltration supports the evolution of a highly immunosuppressive TME in most of the patients.

CD44 is a receptor for hyaluronic acid [58]. High expression of CD44 has been associated with both better [59] and worse outcomes [60], while others did not find an association [58] in ovarian cancer. In this study, we found a higher expression level of CD44 gene in recurrent tumor samples compared to primary tumors, but others have found the opposite in larger cohorts of ovarian cancer samples [59]. CD44 has been shown to be involved in lymphocyte activation and homing [61,62], which could corroborate the increased infiltration of immune cells in the recurrent tumor samples in this study, although more information on the exact function of CD44 is needed before conclusions can be made.

Our findings with increased *CD36* and *CD44* gene expression during disease progression were only confirmed in the patient cohort from Kreuzinger et al. [37] when the patients were stratified for an increase in T cell infiltration from primary to recurrent tumor sample. This suggests that the expression of the two glycoproteins is more related to the degree of T cell infiltration and less to the tumor progression per se.

Indeed, despite progression, we found indications of immune activity in recurrent tumor samples, i.e., infiltration of immune cells and upregulated gene expression related to T cell functions. The concomitant upregulation of genes encoding immunosuppressive molecules such as IDO, PDL1, CEACAM and TGFB indicates that adaptive immune resistance mechanisms are holding the immune system in check. These findings were confirmed in the larger cohort from the study of Kreuzinger and colleagues [37].

In conclusion, we investigated the cancer immunity evolution on a gene and protein expression level in a small cohort of patients with HGSC. We found the tumors to be very heterogeneous, both between primary and recurrent samples and between patients. However, in most patients, we found an increased immune cell infiltration and/or a higher expression of genes related to chemokines, T cell function and immune checkpoints such as TIGIT in recurrent samples compared to primary samples. Our small cohort was uniform with all primary tumors being HGSC and exhibiting a “cold” TME in the primary setting. This condition may explain some of the contradictory results with other studies that included multiple histotypes and/or heterogeneous primary TMEs. Intrinsic molecular features and primary TME background (cold vs. hot) may be a critical designation to consider, as important as histotype, in future studies. The clinically validated gene expression molecular subtyping algorithm for HGSC, PrOTYPE (the Predictor of high-grade-serous Ovarian carcinoma molecular subtype), may provide a reasonable and accessible proxy of TME [63]. A concurrent upregulation of interferon, IDO, PDL1, and TGFB in the TME indicates multiple adaptive immune resistance mechanisms are at play and investigation into multi-target immunotherapy is warranted. The high expression level of the genes encoding LAG3 and TIM3 suggests these immune checkpoint components as possible new targets.

## 4. Materials and Methods

### 4.1. Patient Characteristics

We studied matched primary and recurrent tumor samples from 9 patients with high-grade serous ovarian cancer (HGSC) who after recurrence were enrolled in a phase I clinical trial of ACT (ClinicalTrials.gov Identifier: NCT02482090) carried out at Copenhagen University Hospital, Herlev, Denmark, and previously described in [45]. Primary tissue was collected as formalin-fixed paraffin-embedded (FFPE) blocks from respective hospitals in Denmark. As per the clinical trial protocol, recurrent tissue was obtained after progression on adjuvant chemotherapy and at least one line of treatment for recurrent disease and before the patient received any immunotherapy treatment. Patient and treatment characteristics are provided in Table S1. The scientific use of the patient material was approved by the National Committee on Health Research Ethics (approval no.: 1502266) and the Danish Data Protection Agency. All patients provided written informed consent.

Unfortunately, an FFPE block from patients GY03 was lost after slides were made for IHC staining, which is why this patient was not analyzed with the NanoString technology.

For validation of our findings, we used RNA sequencing data of paired primary and recurrent HGSOC from a cohort of 66 patients (EGAD00010001403), which was published previously by Kreuzinger et al. 2017 [37].

### 4.2. Multicolor Immunohistochemistry/Immunofluorescence

Tumor tissue was analyzed by multicolor immunohistochemistry (IHC) and immunofluorescence (IF), some of which were initially described in [45]. All reagents were obtained from Biocare Medical (Pacheco, CA, USA) unless stated otherwise. Briefly, PDL1/CD163/PD1 combination slides were deparaffinized through xylene and graded alcohols to water then subjected to antigen retrieval with Diva Decloaker. Peroxidased1 and Background Sniper were added to block non-specific staining before a cocktail of anti-PDL1 (clone SP142, Spring Bioscience, Pleasanton, CA, USA) and anti-CD163 (clone 10D6, Biocare) in Da Vinci Green diluent was added for 30 min followed by MACH2 Double Stain # 1 Polymer for 30 min. The signal was detected by adding IP Ferangi blue followed by IP DAB. Slides were then subjected to denaturation at 50 °C for 45 min in an SDS-glycine pH2.0 solution. Slides were then incubated with anti-PD1 (clone EPR4877(2), Abcam, Cambridge, UK) in Da Vinci Green diluent for 30 min followed by MACH2 Rabbit-AP Polymer for 30 min then IP Warp Red Chromogen. Slides were counterstained with a 1:5 dilution of CAT hematoxylin, rinsed, air-dried and coverslipped with Ecomount.

The overall process for staining the TIA1/CD8/CD3 and LAG-3/PD1/CD8 panels was similar. For the TIA1/CD8/CD3 panel, the first IHC round included anti-TIA-1 (clone TIA1, Biocare) and Ferangi Blue chromogen followed by a second round with a cocktail of anti-CD8 (clone C8/144B, Cell Marque, Rocklin, CA, USA) and anti-CD3 (clone SP7, Spring Bioscience) and IP Warp Red Chromogen and Hi Def Yellow Chromogen (Enzo, Farmingdale, NY, USA). For the LAG-3/PD1/CD8 panel, the first IHC round included anti-LAG-3 (clone D2G40, Cell Signaling Technology, Danvers, MA, USA) in Renoir Red diluent followed by Ferengi Blue chromogen, and the second round consisted of anti-PD1 (clone EPR4877(2), Abcam) and anti-CD8 (clone C8/144B, Cell Marque, Rocklin, CA, USA) in DaVinci Green diluent followed by Warp Red chromogen and High Def Yellow chromogen. For the CD20/CD79a panel, one round of staining was performed using a cocktail of CD20 (clone L26, Biocare) and CD79a (clone SP18, Spring Bioscience) followed by Warp Red chromogen and DAB chromogens.

The HLA-I/HLA-II/IDO-1 panel was stained by multicolor IF using OPAL reagents from (Perkin Elmer, Waltham, MA, USA) and the manufacturer’s instructions. Briefly, HLA Class I A, B, C (clone EMR8-5, MBL, Woburn, MA, USA) on OPAL520 was used in round 1, HLA-DP, DQ, DR (clone CR3/43, Affinity Bioreagents, Golden, CO, USA) on OPAL650 in round 2, and IDO-1 (clone SP260, Spring Bioscience) on OPAL570 in round 3. Primary antibodies were detected by suitable MACH2 polymers (Biocare).

### 4.3. Imaging and Quantification

Slides were imaged using the Vectra 2 multispectral imaging system (Perkin Elmer). Ten 20× images per slide (with at least 90% tissue content) were analyzed using InForm image analysis software (Perkin Elmer). Cell densities were calculated, and H scores were determined for IDO-1, HLA-I and HLA-II. Each panel was scored using 5 algorithms with varying levels of permissiveness. The results were confirmed with visual validation before average scores were reported.

### 4.4. NanoString Specimen Processing

Matched primary and recurrent tumor samples from eight patients with ovarian cancer were investigated with NanoString technology. Briefly, specimens were processed on unstained FFPE sections on glass slides. A pathologist reviewed and marked an adjacent H&E slide to identify regions of high tumor content without the removal of infiltrating stroma. Unstained sections were then manually macrodissected with a scalpel using the marked H&E as a guide. Total RNA was extracted from the dissected FFPE tissue using a modified version of the Qiagen miRNeasy FFPE extraction kit (Qiagen, Hilden, Germany) described previously [51]. Total RNA (500 ng) was mixed with NanoString codeset and codeset plus reagents and processed following the manufacturer’s protocol recommendation (NanoString XT gene expression protocol; NanoString). For biopsy samples with less than 500 ng total RNA recovered we applied all available total RNA in 5 μL (range 100–500 ng). Following overnight hybridization, samples were washed and transferred to NanoString cartridges using the nCounter Prep Station and imaged at maximum FOV on a nCounter FLEX Digital Analyzer (NanoString).

Data from output RCC files were extracted using nSolver software (NanoString) and quality assessment (QA) as previously described [51,55]. Samples passing QA were normalized to 38 housekeeping genes to balance out the different levels of mRNA caused by the age and manufacture of the FFPE blocks and analyzed with advanced analysis with default settings.

A heatmap was generated using unsupervised clustering from the normalized data and scaled to provide all genes equal variance. Furthermore, log2 fold change (log2FC) of gene expression levels was calculated for each patient and visualized in a heatmap created with Graphpad Prism 8 software.

Gene expression heatmaps, clustering analyses and signature pathway scores were generated with nSolver software.

Differential gene expression was calculated using a generalized linear model from the EdgeR package for R [64]. The primary samples were used as baseline samples. For the volcano plot, each gene was plotted by −log10(FDR) and log10(fold change).

Correlation of expression of selected genes was examined with linear regression analysis and tested for significance with a Spearman correlation test in Graphpad Prism 8 software and tested for significant difference in expression between primary and recurrent tissue with Wilcoxon matched-pair signed rank test in Graphpad Prism 9 software.

### 4.5. Flow Cytometry Analysis of TILs from Recurrent Tumors

TILs were isolated and expanded from recurrent tumor biopsies using high dose IL-2 (6000 IU/mL) containing media as previously described [45]. The TILs were manufactured for the purpose of treating the patients with TIL-based ACT. TILs were thawed and stained the same day with the following panel, NiR live dead, CD3-BV786, CD4-BV510, CD8-APC-R700, LAG3-FITC, TIM3-BV711, TIGIT-BV650, and PD1-PE-594 dazzle. The cells were acquired using a Quanteon (ACEA) flow cytometer and analyzed with NovoExpress software version 1.4.1.

## 5. Conclusions

Our findings suggest that ovarian cancer evolves into a more inflamed tumor type susceptible to immunotherapy treatment. However, since ovarian cancer has not shown encouraging responses to immunotherapy yet, we propose new targets such as the immune checkpoints LAG3 and TIM3 based on our gene expression analyses. Further, we observed an increased gene expression of two other immune checkpoints, TIGIT and CTLA4 in the TME, which imply that these could be taken into account as targets when treating patients with recurrent disease. We believe that our study identifies interesting targets as an alternative to the PD1/PDL1 targeting antibodies.

## Supplementary Materials

The following are available online at https://www.mdpi.com/2072-6694/12/12/3828/s1. Figure S1: PDL1 expression on macrophages. Figure S2: Stromal infiltration in the TME. Figure S3: NanoString analysis—CD36 expression. Figure S4: NanoString analysis—Granzyme and CD137 expression. Figure S5: Validation cohort. Figure S6: Flow cytometry analysis of immune checkpoint expressions on CD8+ TILs. Table S1: Patient Characteristics.
