# Supplementary material for: Changes in the Tumor Immune Microenvironment during Disease Progression in Patients with Ovarian Cancer
Source: Cancers (Basel). 2020 Dec 18;12(12):3828. doi: 10.3390/cancers12123828 (PMC7767114; doi:10.3390/cancers12123828)
Supplement: Supplementary file 1 [file cancers-12-03828-s001.zip › cancers-996328-supplement.docx]

Supplementary Materials

Changes in the Tumor Immune Microenvironment during Disease Progression in Patients with Ovarian Cancer

Marie Christine Wulff Westergaard, Katy Milne, Magnus Pedersen, Thomas Hasselager, Lars Rønn Olsen, Michael S. Anglesio, Troels Holz Borch, Mia Kennedy, Gillian Briggs, Stacey Ledoux, Caroline Kreuzinger, Isabel von der Decken, Marco Donia, Dan Cacsire Castillo-Tong, Brad H. Nelson and Inge Marie Svane


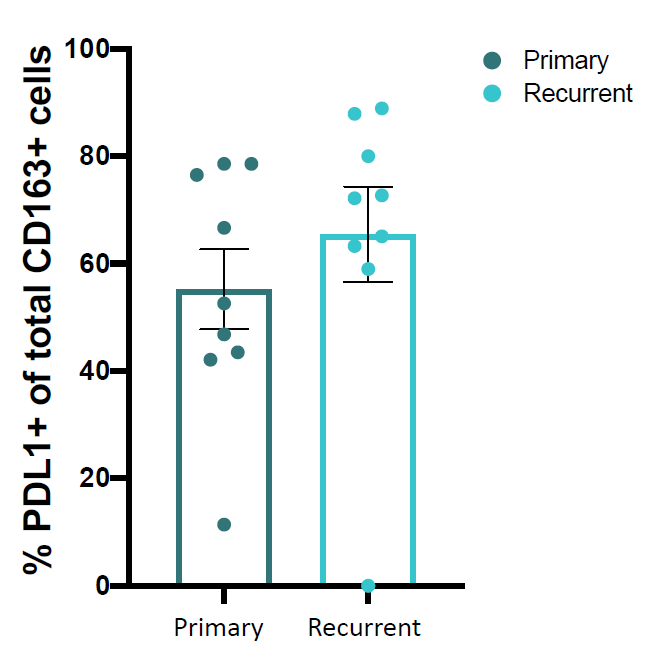


**Figure S1:** PDL1 expression on macrophages. Bar chart represents the mean percentage with SEM of macrophages expressing PDL1 out of total amount of infiltrating macrophages and dots represent each patient.


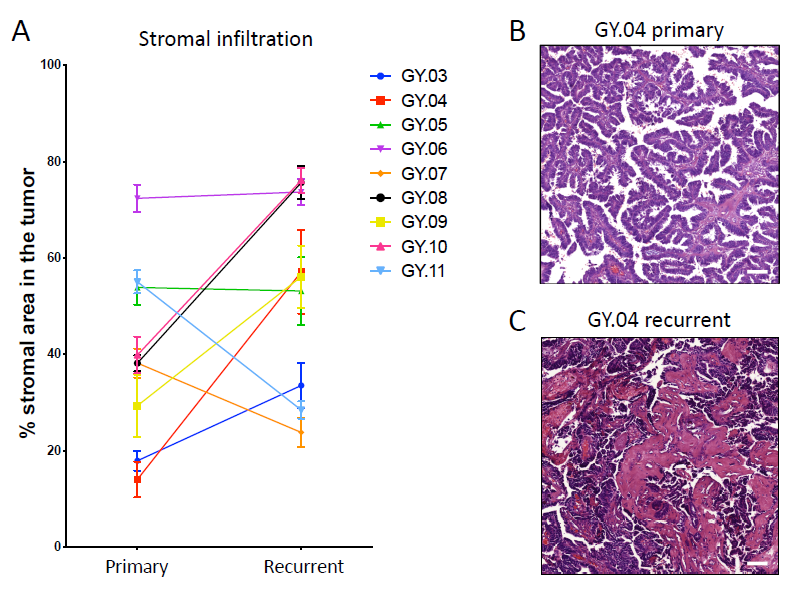


**Figure S2:** Stromal infiltration in the TME. (A) Illustrating the quantity/portion of the stromal compartment in the TME in the primary and recurrent tumor samples. (B and C) Example of stromal

infiltration in the primary (B) and recurrent (C) tumor tissue in patient GY.04. The white scalebar is 100 um.


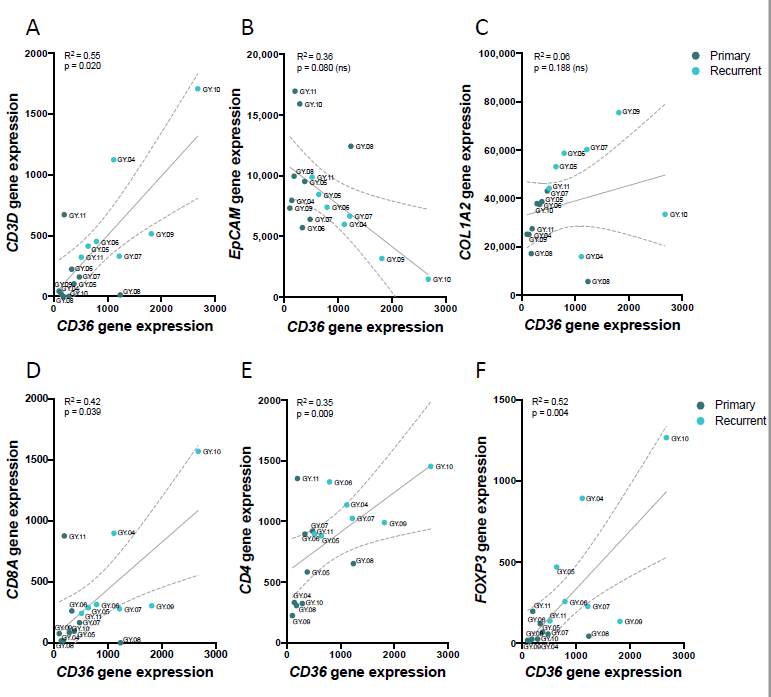


**Figure S3:** NanoString analysis – CD36 expression. Correlation between CD36 gene expression and CD3 (A), EpCAM (B), COL1A2 (C), CD8A (D), CD4 (E), and FOXP3 (F) gene expression in all tumor samples. The data are presented with R2 values and Spearman correlation test p values.


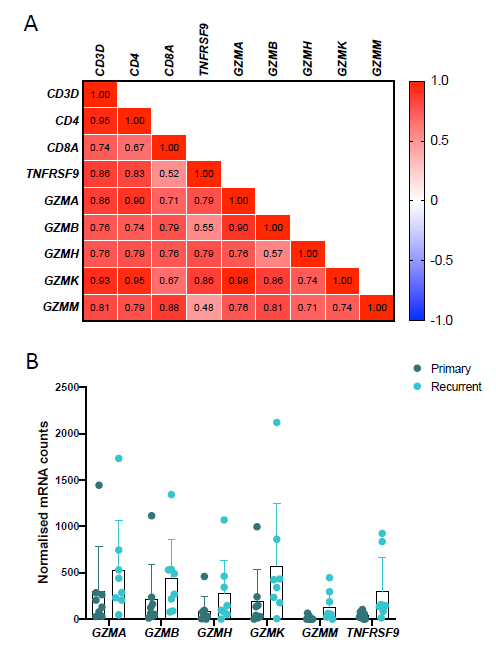


**Figure S4:** NanoString analysis – Granzyme and CD137 expression. (A) Spearman correlation matrix with r values, from log2 fold change values of the expression of granzymes and CD137 encoding genes

from the primary and recurrent tumor samples. Red indicates a strong positive correlation, blue indicates strong negative correlation and white indicates no correlation. (B) Gene expression levels of

granzymes and CD137 encoding genes in primary and recurrent tumor. Statistical difference between primary and recurrent was performed with Multiple Wilcoxon test in Graphpad prism 9 software. No statistical difference was found when using (FDR). GZMA: p=0.315, GZMB: p=0.303, GZMH: p=0.295, GZMK: p=0.221, GZMM: p=0.221, TNFRSF9: p=0.047.


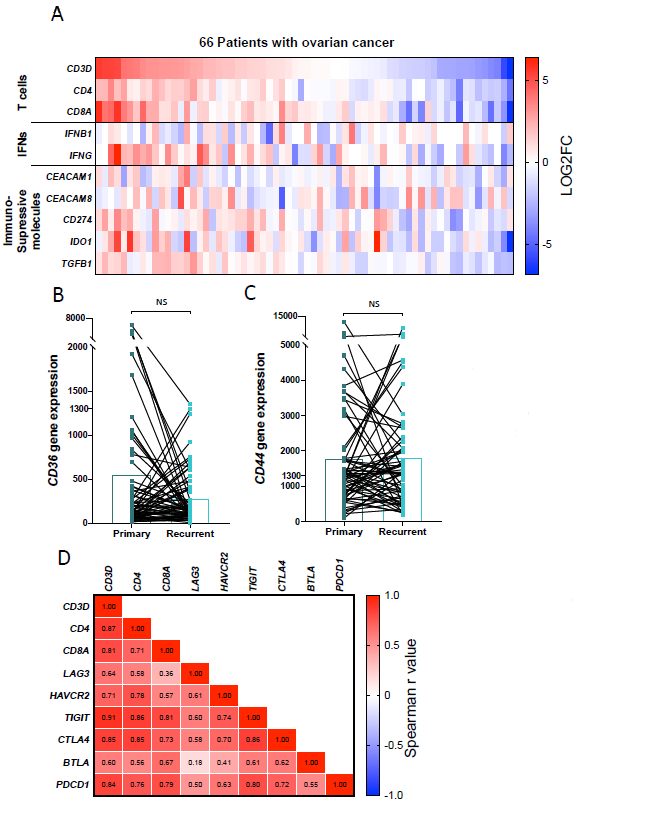


**Figure S5:** Validation cohort. All following figures shows RNA seq data from primary and recurrent

tumor from a cohort of 66 patients previously described in Kreuzinger et al. [34] (**A**) Heatmap showing the log2 fold change values of the expression of selected genes from primary and recurrent tumor samples. Red indicates high expression and blue indicates low expression. Gene expression of CD36

(**B**) and CD44 (**C**) in primary and recurrent tumor. (**D**) Spearman correlation matrix with r values of T cell maker genes and immune checkpoint genes. Red indicates a strong positive correlation, blue indicates strong negative correlation and white indicates no correlation.


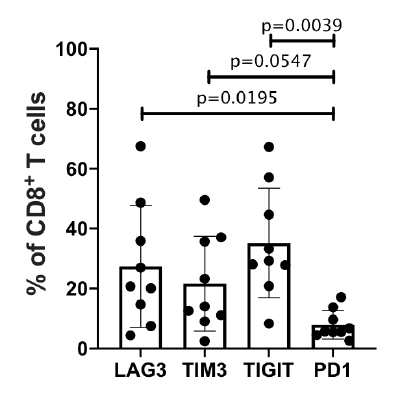


**Figure S6:** Flow cytometry analysis of immune checkpoint expressions on CD8+ TILs. TILs isolated and expanded from the recurrent tumor biopsies were analyzed with flow cytometry for the expression of immune checkpoints. The figure shows the percentage of immune checkpoint positive CD8+ T cells out of total CD8+ T cell fraction. Dots represents each patients, bars represent mean +SD. Data were tested for statistical significance using Wilcoxon matched-pairs signed rank test, p-values are displayed on figure.
